# Ablating Left-sided Atrioventricular Accessory Pathways in Young Patients with Persistent Left Superior Vena Cava: Start in the Coronary Sinus

**DOI:** 10.19102/icrm.2025.16121

**Published:** 2025-12-15

**Authors:** Soham Dasgupta, Christopher Johnsrude

**Affiliations:** 1 Division of Pediatric Cardiology, Department of Pediatrics, Norton Children’s Hospital, University of Louisville, Louisville, KY, USA

**Keywords:** Ablation, coronary sinus, pediatrics, persistent left superior vena cava

## Abstract

While hemodynamically insignificant, a persistent left superior vena cava (PLSVC) draining to the coronary sinus (CS) may have important implications during an electrophysiology study and catheter ablation. Specifically, ablation of left-sided accessory pathways (APs) poses a special challenge secondary to the potential distortion of the mitral valve annulus (MVA) and the possibility of the AP comprising a discrete epicardial trunk involving CS musculature. Ablation of such pathways is more likely accomplished from within the CS rather than on the MVA after a transseptal puncture. We describe two pediatric cases with a PLSVC draining to a dilated CS in whom successful ablation of left-sided APs was accomplished from within the CS after failed ablation attempts on the MVA. Initial mapping and ablation in the CS after a careful evaluation of the coronary artery proximity may obviate the need for a transseptal puncture with its potential challenges.

## Introduction

Persistent left superior vena cava (PLSVC) is a congenital anomaly of the thoracic venous system resulting from abnormal embryological regression, which usually drains into the right atrium via a patulous coronary sinus (CS).^1^ This anomaly does not impose significant hemodynamic consequences and is often discovered incidentally on cardiovascular imaging or during invasive cardiovascular procedures.^1^ However, a PLSVC may pose procedural challenges for patients presenting for catheter ablation to treat supraventricular tachycardia (SVT),^2^ including anatomic distortions affecting electrophysiological (EP) mapping within the triangle of Koch in patients with atrioventricular (AV) nodal re-entry tachycardia (AVNRT),^3^ atypical anatomy and courses of AV accessory pathways (APs) in those with AV re-entry tachycardia (AVRT),^4^ and potentially challenging transseptal puncture procedure related to congenital septal anatomy.^2^

While successful catheter ablation of left-sided APs on the mitral valve annulus (MVA) has been described in adult patients with PLSVC, there are limited reports of catheter ablation within the PLSVC^5,6^ in children, some of whom had concomitant congenital heart defects (CHDs). Herein, we describe successful catheter ablation within the PLSVC to treat two young patients with AVRT and CHD, after unsuccessful ablation had been performed on the MVA via a transseptal approach.

This study was approved by the institutional review board at Norton Children’s Hospital.

## Case presentations

### Case 1

**Figure 1:**
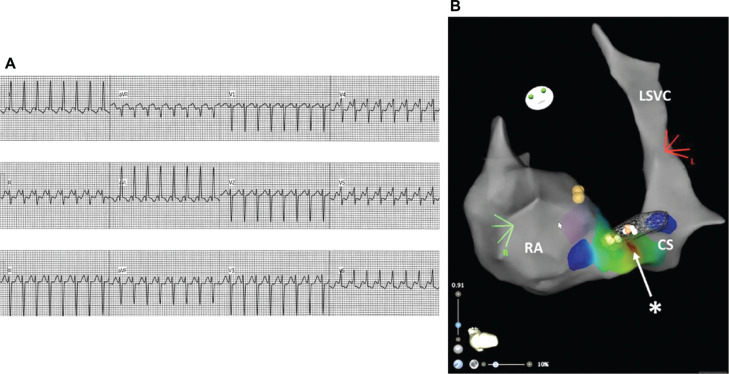
Atrioventricular re-entry tachycardia due to a concealed left-sided accessory pathway in a 9-year-old girl with multiple ventricular septal defects and persistent left superior vena cava draining to a dilated coronary sinus (CS). **A:** 12-lead electrocardiogram of supraventricular tachycardia. **B:** Three-dimensional maps demonstrate the geometry of the right atrium, left superior vena cava, and dilated CS; location of the bundle of His (yellow tags); and early atrial activation in the CS during inducible SVT (area in red). The successful ablation site in the CS (*) is somewhat close to two prior unsuccessful radiofrequency ablation sites (white tags) previously placed on the mitral valve annulus (in mesh) via a transseptal approach. *Abbreviations:* CS, coronary sinus; LSVC, left superior vena cava; RA, right atrium.

A 9-year-old girl born with multiple restrictive ventricular septal defects (VSDs) and Wolff–Parkinson–White (WPW) syndrome had been previously treated with flecainide. After her WPW syndrome appeared to have resolved spontaneously and flecainide was discontinued, she was lost to follow-up until intermittent pre-excitation was recorded on continuous inpatient telemetry when she was admitted for a prolonged febrile illness. She was referred back for EP consultation, and the family requested an EP study and possible catheter ablation after shared medical decision-making. Her weight at the time of the EP study was 47 kg. Under general anesthesia and via percutaneous access to the right femoral vein (RFV), three-dimensional (3D) cardiac geometries created using an EZ Steer D/F mapping-ablation catheter and CARTO3 system (Biosense Webster, Diamond Bar, CA, USA) demonstrated a PLSVC draining to a dilated CS. Preoperative echocardiograms did not clearly demonstrate the presence of a PLSVC. An additional intracardiac diagnostic EP catheter was advanced via the RFV, and a transesophageal EP catheter was placed to record far-field left atrial and ventricular electrograms when a sheath wire would not advance in the left femoral vein. Initial EP testing showed no evidence of ventricular pre-excitation at baseline or during administration of adenosine or isoproterenol but readily induced sustained AVRT due to a left-sided AP **(Figure 1A)**. Absent a patent foramen ovale, the transseptal puncture procedure uneventfully gained access to the left atrium under fluoroscopy (exposure for 2.1 min), followed by systemic heparinization (activated clotting time target, 300 s). Mapping retrograde atrial activation during right ventricular (RV) pacing was contaminated by robust retrograde AV nodal conduction, and we could not obtain stable ventricular capture from a lateral coronary venous tributary to provide stable left ventricular pacing.

Therefore, retrograde atrial activation during AVRT was mapped along the MVA, and an optimal site was found at a left posterior location **(Figure 1B)**. Radiofrequency (RF) catheter ablation (40 W, target temperature of 50°C) was performed during SVT, terminating tachycardia with block in the AP in <2 s. However, tachycardia was readily re-induced despite additional RF applications at this initial site and consolidations in proximity. The MVA was remapped during SVT, and early retrograde atrial activation on the MVA was now ~5 mm lateral to the initial map **(Figure 1B)**. RF applications at this new site had similar outcomes, with SVT termination in the AP, followed by SVT inducibility.

The initial 3D map of SVT within the CS was compared to those created on the MVA **(Figure 1B)**. The EZ Steer catheter was repositioned into the CS, and when the catheter tip reached the optimal site, SVT frequently terminated with block in the AP; notably, such “bumping” did not occur when mapping the MVA. We suspected that the AP consisted of a narrow epicardial trunk associated with CS musculature that branched to multiple left atrial insertions. Prior to ablating within the CS, selective coronary angiography showed no coronary artery branches within 5 mm of the optimal CS ablation site.^7^ With RF ablation at this site (20 W, target, 50°C) during SVT, ventriculoatrial (VA) block was seen at 0.9 s, and a 30-s application was completed **(Figure 1B)**. After three consolidation applications for 30–60 s each, the VA block persisted at baseline and with administration of adenosine and isoproterenol, and SVT remained non-inducible over the next 60 min. She was discharged home after an uneventful recovery and has done well over the 6-month follow-up. Serial electrocardiograms (ECGs) have not shown worrisome ST/T changes or pre-excitation. She will resume routine follow-up with her pediatric cardiologist for her restrictive VSDs.

### Case 2

An 18-year-old girl with complex CHD was doing well hemodynamically after surgical repair of coarctation of the aorta, perimembranous VSD, and subaortic membrane; her PLSVC draining to the CS had required a modified approach for a safe cardiopulmonary bypass. In addition, she had WPW syndrome managed with chronic flecainide therapy. When she developed frequent breakthrough AVRT, she was referred for catheter ablation. Her weight at the time of the study was 80 kg. At the EP study, 3D geometries using an EZ Steer catheter and the CARTO3 system confirmed her right heart anatomy and PLSVC draining to a dilated CS. EP testing using two additional intracardiac diagnostic EP catheters showed no evidence of ventricular pre-excitation, but it did induce sustained orthodromic AVRT conducted with a baseline right bundle branch block pattern. EP mapping showed early retrograde atrial activation on distal CS electrodes consistent with a concealed left lateral AP. Transseptal puncture using fluoroscopy (3 min) accessed the left heart, followed by systemic heparinization. Initial EP mapping along the MVA during SVT demonstrated diffuse early atrial activation. RF ablations (50 W, 60°C) targeting the earliest site on the MVA had no effect on SVT. VA conduction via the AP remained unchanged on CS recordings, but SVT became progressively difficult to induce, and then was only non-sustained. Subsequent 3D maps of retrograde AP conduction on the MVA during pacing from the RV apex and outflow tract again demonstrated rather diffuse atrial activation, with the earliest atrial activation shifting more atrial and posterior to sites targeted for ablation.

**Figure 2:**
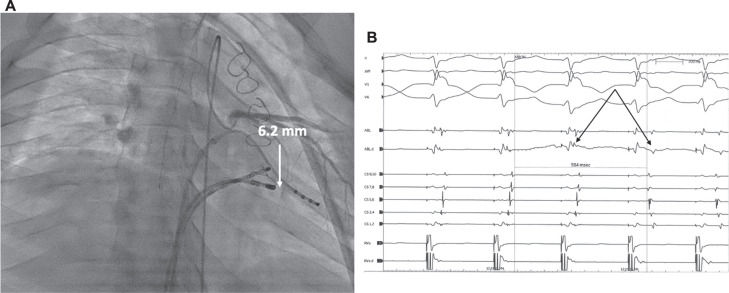
Atrioventricular re-entry tachycardia due to a left-sided accessory pathway in an 18-year-old girl with postoperative complex congenital heart disease and pre-testing right bundle branch block pattern on electrocardiography. After failed radiofrequency ablation on the mitral valve annulus, the radiofrequency catheter was advanced in the coronary sinus, where the earliest retrograde atrial activation was recorded. **A:** Left main coronary angiography shows that the optimal site for ablation is 6.2 mm from the closest branch. **B:** With radiofrequency ablation at this site during ventricular pacing, ventriculoatrial block in the accessory pathway occurs in <1 s (arrows).

We repositioned the EZ Steer catheter within the dilated CS and advanced a diagnostic EP catheter via the transseptal sheath for left ventricular apical pacing closer to the AP. EP mapping of atrial activation within the CS during left ventricular pacing demonstrated a discrete area on the posterolateral floor of the CS, near the diffuse atrial activation on the MVA. Selective coronary angiography confirmed no coronary branches within 5 mm of the optimal ablation site **(Figure 2A)**.^7^ The EZ Steer catheter was exchanged for an irrigated QDOT™ RF catheter (JNJ Medtech, New Brunswick, NJ, USA), and ablation (20 W, 47°C) during ventricular pacing led to immediate loss of retrograde AP conduction **(Figure 2B)**; a 30-s lesion was placed here. After four consolidation applications in this area (target ablation index, 400), EP testing showed no evidence of AP conduction or inducible SVT at baseline or with adenosine or isoproterenol. She recovered uneventfully, and ST/T patterns on ECG remained stable. She has done well during her 3-month follow-up.

## Discussion

Patients with AVRT and PLSVC draining to a dilated CS often have an AV AP that is left-sided, and atypical anatomy even in those without additional congenital cardiac defects can introduce challenges to successful ablation, including intracardiac access, complex AP anatomy, accurate EP mapping, and tip–tissue stability for ablation.^4^ Our experience with two young patients highlights that the AP can involve the CS musculature, consisting of a common trunk within the CS that branches to multiple left atrial insertions, and successful and safe ablation can be performed within the CS without requiring a transseptal puncture to map and target sites on the MVA. Additionally, as encountered in case 1, it may be that some complex fibers require ablation from the epicardial surface (within the CS) combined with an endocardial approach at the atrial or ventricular side of the MVA.

A PLSVC is the most common congenital thoracic venous anomaly, typically drains to the right atrium via a dilated CS, and presents no significant hemodynamic implications except pertaining to surgical or catheter-based interventions.^8,9^ The LSVC develops from the proximal left sinus horn of the sinus venosus at around 7–8 weeks of gestational age,^2^ persisting when there is incomplete embryological regression. Around the same time, fibrous separation between the atria and ventricles occurs, and incomplete separation is responsible for residual myofibers left bridging an AV annulus to form AV AP(s) that can provide a substrate for subsequent episodes of AVRT. The interplay between these two principal embryological events is likely why the APs in patients with AVRT and PLSVC are usually left-sided and have a complex anatomy.^2,4,6,10–13^ Of note, PLSVCs are found incidentally in patients with otherwise structurally normal hearts but also in patients with diverse CHDs. APs involving the CS musculature in patients without PLSVC are relatively uncommon but have been well described, and treatment success may require ablation within the CS or middle cardiac vein.^12^

There are several prior reports of catheter ablation to treat AVRT in adults with PLSVC,^2,4,5^ including ablation of left-sided APs in the CS. These cases can be difficult, requiring extensive endocardial mapping and involving multiple ablation applications on the MVA and targeting CS musculature.^12^ Sometimes proceduralists have to be creative, for example, using a retrograde CS approach from the left internal jugular vein to ablate within the CS when the PLSVC is accompanied by CS ostial atresia.^2^ Similar to our experience, one report described failed ablation on the MVA followed by successful ablation within the CS.^5^ This case in particular described switching to a 3D anatomic mapping for careful localization and visualization within the dilated CS secondary to catheter instability, a technique we employed from the beginning in both our cases. It is not surprising that there is a higher post-ablation risk of AVRT recurrence in patients with PLSVC.^13^ To the best of our knowledge, there is only one reported pediatric case of ablation of a left-sided AP within the CS.^6^ In that report, ablation of a posteroseptal antegrade-only AP responsible for ventricular pre-excitation was performed in an 11-year-old girl with d-transposition of the great arteries s/p arterial switch surgery. The authors first attempted unsuccessful ablation on the MVA, similar to our cases, but were able to eliminate the AP when ablating within the CS.

Our experience suggests that it is reasonable to start mapping left-sided APs in patients with a PLSVC by focusing on the CS proper, with a detailed 3D mapping to attempt to uncover optimal ablation target(s). If such mapping indicates a discrete target, starting with ablation in the CS is not unreasonable. If successful, this approach obviates the need to perform a transseptal puncture, which could be challenging due to a dilated CS ostium and atypical atrial septal contours, systemic heparinization, and delivery of multiple ablation applications in the left heart. Whether or not to perform coronary angiography prior to ablation in the CS will remain an individual decision.

## Conclusion

Pre- and intraprocedural evaluation for a PLSVC to a dilated CS is important in pediatric patients with AVRT undergoing an EP study and catheter ablation and carries significant implications. When a PLSVC is encountered, dense 3D EP mapping within the CS may identify an optimal ablation target as seen in our cases, comprising a discrete epicardial “trunk” involving CS musculature, branching to multiple atrial insertions near the MVA. Optimal ablation target(s) within the CS may be remote from epicardial coronary arteries and safely and effectively ablated within the CS. Furthermore, this approach may obviate the need for a transseptal puncture with its potential challenges due to distorted atrial septal anatomy, mitigating the need for higher levels of systemic heparinization and avoiding ablation applications in the left heart.
